# Data Sharing Policy for Balkan Medical Journal

**DOI:** 10.4274/balkanmedj.galenos.2019.2019.2.001

**Published:** 2019-02-28

**Authors:** Okan Çalıyurt, Zafer Koçak

**Affiliations:** 1 Department of Psychiatry, Trakya University School of Medicine, Edirne, Turkey; 2 Department of Radiation Oncology, Trakya University School of Medicine, Edirne, Turkey

In 2004, the International Committee of Medical Journal Editors (ICMJE) announced a statement on clinical trial registration (1). According to the statement, a clinical trial is defined as any research project that prospectively assigns human subjects to intervention or comparison groups to study the cause-and-effect relationship between a medical intervention and a health outcome. Thereafter, this policy was well adopted by the ICMJE member journals.

In recent years, data sharing has become increasingly important in scientific journals, and ICMJE took a step forward in 2017 to make data sharing mandatory in clinical trials for the member journals (2).

Data sharing provides transparency and openness. In addition, it helps expand our horizons in science. With the sharing of data, it is possible to surpass research and discoveries beyond the presentation of scientific data in literature through interpretation only by the authors. Replication of studies helps in assessing the validity of previously published studies. The most accurate way of replication of studies can be via sharing of data, materials, and protocols.

Data sharing should not be interpreted as the sharing of the authors’ valuable work without benefitting from it. With the sharing of data, the information will become more reliable. The possibility of obtaining a citation will increase; the contribution to literature will also widen. Furthermore, it will be possible to enrich the data by combining it with other similar data sets. This will in turn contribute to science through interpretation, combination, and study of shared data by other scientists. The authors will benefit by data sharing through its positive impact on grant providers and financial supporters.

Balkan Medical Journal is aware of the importance of data sharing to increase openness and transparency and to provide a richer and reliable content to scientific literature. However, Balkan Medical Journal follows the recommendations of the ICMJE (3). As of January 2019, Balkan Medical Journal has declared compliance with the ICMJE’s data sharing statement because it has accepted the benefits of data-sharing as outlined above and is a follower of ICMJE. Please refer to the section of instructions to the authors of Balkan Medical Journal.

Finally, the standards of data sharing remain unclear. The mode of transmission of data and the characteristics of the shared data will be determined over time. Balkan Medical Journal will follow-up the relevant issues to update the data-sharing policies and continuously inform the authors, reviewers, and editors.
